# COTI-2, a novel small molecule that is active against multiple human cancer cell lines *in vitro* and *in vivo*

**DOI:** 10.18632/oncotarget.9133

**Published:** 2016-05-02

**Authors:** Kowthar Y. Salim, Saman Maleki Vareki, Wayne R. Danter, James Koropatnick

**Affiliations:** ^1^ Critical Outcome Technologies Inc., London, Ontario, Canada; ^2^ Cancer Research Laboratory Program, Lawson Health Research Institute, London, Ontario, Canada; ^3^ Department of Microbiology and Immunology, Western University, London, Ontario, Canada; ^4^ Department of Pathology, Western University, London, Ontario, Canada; ^5^ Department of Oncology, Western University, London, Ontario, Canada; ^6^ Department of Physiology and Pharmacology, Western University, London, Ontario, Canada

**Keywords:** COTI-2, CHEMSAS, targeted-therapy, cancer, small molecule

## Abstract

Identification of novel anti-cancer compounds with high efficacy and low toxicity is critical in drug development. High-throughput screening and other such strategies are generally resource-intensive. Therefore, *in silico* computer-aided drug design has gained rapid acceptance and popularity. We employed our proprietary computational platform (CHEMSAS^®^), which uses a unique combination of traditional and modern pharmacology principles, statistical modeling, medicinal chemistry, and machine-learning technologies to discover and optimize novel compounds that could target various cancers. COTI-2 is a small molecule candidate anti-cancer drug identified using CHEMSAS. This study describes the *in vitro* and *in vivo* evaluation of COTI-2. Our data demonstrate that COTI-2 is effective against a diverse group of human cancer cell lines regardless of their tissue of origin or genetic makeup. Most treated cancer cell lines were sensitive to COTI-2 at nanomolar concentrations. When compared to traditional chemotherapy or targeted-therapy agents, COTI-2 showed superior activity against tumor cells, *in vitro* and *in vivo*. Despite its potent anti-tumor efficacy, COTI-2 was safe and well-tolerated *in vivo.* Although the mechanism of action of COTI-2 is still under investigation, preliminary results indicate that it is not a traditional kinase or an Hsp90 inhibitor.

## INTRODUCTION

Recent advances in high-throughput cancer genome sequencing such as whole genome sequencing and functional screening using RNA interference have revealed exploitable characteristics of cancer cells that could be targeted in cancer treatment [1]. Therefore, novel small molecules are designed or screened to target genetic addictions, susceptibilities, and dependencies of cancer cells resulting in the transition from cytotoxic chemotherapy to the targeted-therapy era. However, discovering a novel and effective drug can be extremely challenging since not all discovered biological targets are druggable [2]. Moreover, developing a drug candidate either through relatively unbiased high-throughput screening (HTS) processes that automate laboratory techniques, or hypothesis-driven research (in the case of molecules targetable by rational design of therapeutic agents), is often challenging and can take years [3]. *In silico* drug design that simulates HTS in combination with elements of rational design has played a more prominent role in the identification of therapeutically-important small molecules in the past three decades [4]. The advantage of computer-aided drug design over HTS is that, unlike unbiased methods, it is capable of ranking candidate therapeutic compounds to allow selection of a manageably small number for screening in the laboratory [5]. In addition, the inclusion of rational elements in the ranking process (for example, selection of the most effective and least toxic structures from existing therapeutic compounds) reduces both time and cost required for preclinical development [6]. However, despite the inefficiency and the high cost associated with virtually all HTS strategies, they remain common in the drug development process. Therefore, computational technologies that can precisely identify and predict structures with desired inhibitory effects and low toxicity are of utmost value to the modern process of drug development [4].

We applied a novel and proprietary computational platform called CHEMSAS^®^ that uses a unique combination of traditional and modern pharmacology principles, statistical modeling, medicinal chemistry, and machine-learning technologies to discover, profile, and optimize novel compounds that could target various human malignancies. At the centre of the CHEMSAS platform is a hybrid machine-learning technology that can find, profile, and optimize novel targeted lead compounds. It can also find novel uses for known compounds and solve problems with existing or potential drugs stored in its database. The CHEMSAS platform is supported by “Chembase”, which is a proprietary powerful database comprised of over a million known compounds with associated laboratory data covering a wide variety of biological and pharmacokinetic targets.

Using the CHEMSAS platform, we developed 244 molecular descriptors for each candidate therapeutic compound. For example, we assessed molecular properties relating to a candidate compound's therapeutic efficacy, expected human toxicity, oral absorption, cumulative cellular resistance, and its kinetics. In some instances, comparative properties relating to commercially relevant benchmark compounds were also assessed. Potential lead compounds were then selected from the candidate library using a proprietary decision-making tool designed to identify candidates with the optimal physical chemical properties, efficacy, and ADMET properties (absorption, distribution, metabolism, excretion, and toxicity) according to a pre-determined set of design criteria.

COTI-2, the lead compound selected from the candidate library of up to 10 novel compounds on multiple scaffolds optimized for the treatment of various cancers, was synthesized for further development. The preclinical development of COTI-2 included the *in vitro* and *in vivo* evaluation of the compound against a variety of cancer cell lines. This testing acts as further validation of our proprietary platform. In this study, we investigated the anti-cancer effects and conducted a preliminary exploration of the mechanism of action of COTI-2. Our results show that COTI-2 is highly efficacious against multiple cancer cell lines from a broad range of human cancers both *in vitro* and *in vivo*. Furthermore, it demonstrated a low toxicity profile in mice and better efficacy when compared to standard chemotherapeutic and targeted-therapeutic agents. Early mechanism of action studies demonstrate that COTI-2 is not a traditional kinase inhibitor nor is it an Hsp90 protein inhibitor and that it causes cancer cell death via apoptosis.

## RESULTS

### COTI-2 inhibits the growth of various cancer cell lines *in vitro*

**Figure 1:**
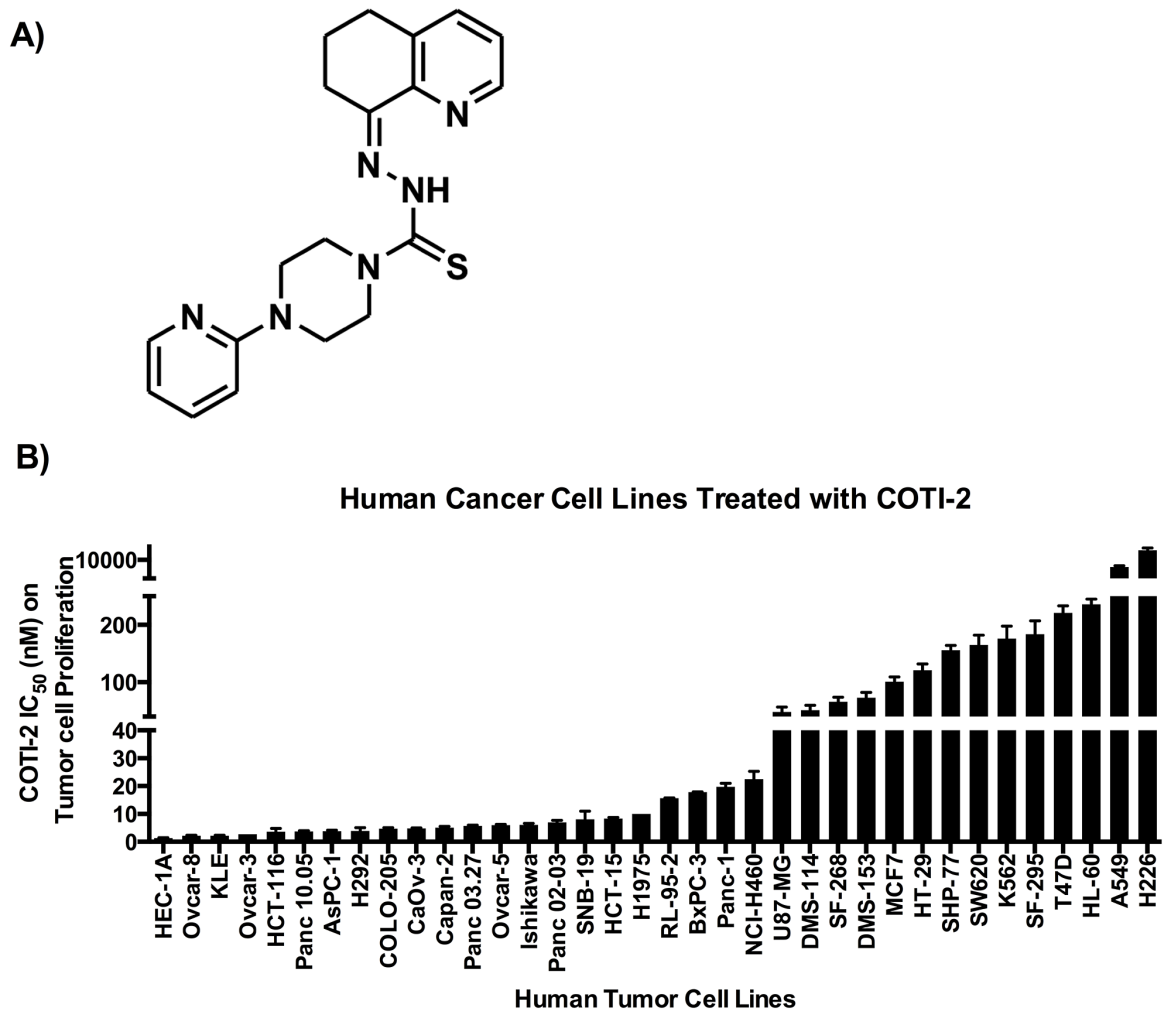
**A.** COTI-2, a third generation thiosemicarbazone, was designed using the CHEMSAS computational platform. **B.** Human cancer cell lines were treated with COTI-2. Tumor cell proliferation was examined 72 h after treatment with COTI-2. The IC_50_ values were calculated from four independent experiments. Error bars indicate SEM.

CHEMSAS is a novel *in silico* machine learning process that predicts target biological activities from molecular structure. We used CHEMSAS to design COTI-2, a third-generation thiosemicarbazone engineered for high efficacy and low toxicity (Figure 1A). We tested the efficacy of COTI-2 against a diverse group of human cancer cell lines with different genetic mutation backgrounds. COTI-2 efficiently inhibited the proliferation rate of all the tested cell lines following 72 h of treatment (Figure 1B). Most cell lines showed nanomolar sensitivity to COTI-2 treatment, regardless of the tissue of origin or genetic makeup.

### COTI-2 is more effective at inhibiting tumor cell proliferation than cetuximab and erlotinib

**Figure 2:**
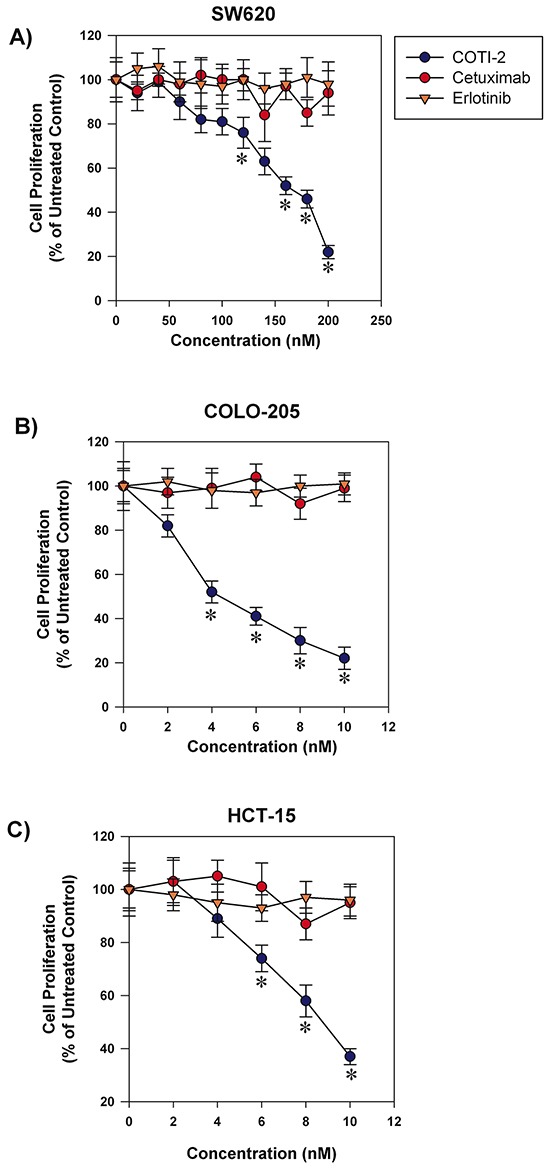
COTI-2 is significantly more effective than cetuximab or erlotinib in inhibiting colorectal cancer cell line proliferation Human colorectal cancer cell lines **A.** SW620, **B.** COLO-205, and **C.** HCT-15 were treated with varying concentrations of cetuximab, erlotinib, or COTI-2. Tumor cells were allowed to proliferate for 96 h before cell viability was examined using alamarBlue assay. All data points indicate the mean of five independent measures of viability ± SEM. *Significantly different from cells treated with cetuximab or erlotinib using a Student's *t*-test (*p* < 0.05).

Targeted-therapy drugs are often designed to have lower toxicity towards normal cells [7]. Agents such as cetuximab and erlotinib are used to treat various types of cancers including colorectal cancer, head and neck squamous cell carcinoma, non-small cell lung cancer (NSCLC), and pancreatic cancer [8–11]. These drugs are designed to specifically inhibit epidermal growth factor receptors that are often over-expressed and/or mutated in the aforementioned cancers [8, 12]. COTI-2 showed potent anti-proliferative activity against a broad collection of human cancer cell lines including some malignancies for which cetuximab and/or erlotinib are approved treatments. We therefore compared the anti-proliferative capacity of COTI-2 against both cetuximab and erlotinib in three human colorectal cancer cell lines. As shown in Figure 2, COTI-2 was significantly more effective at inhibiting tumor cell proliferation than either cetuximab or erlotinib in all three cell lines (COLO-205, HCT-15, and SW620). Notably, all three lines were insensitive to growth inhibition (cytostasis, cytotoxicity, or a combination of the two) to any degree in response to low concentrations of cetuximab and erlotinib, but highly sensitive to even low doses of COTI-2. Similar results were observed in the H292 and H1975 human NSCLC cell lines that suggest these cell lines are more sensitive to COTI-2 than erlotinib (Supplementary Table S1).

### Human glioblastoma cells are more sensitive to COTI-2 than to cisplatin or BCNU

**Figure 3:**
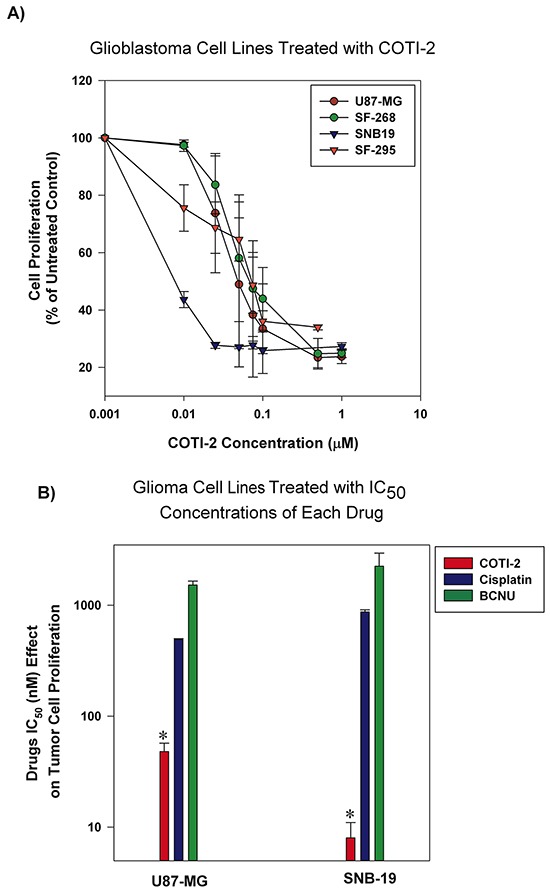
Human glioblastoma cell lines are sensitive to COTI-2 treatment **A.** Human glioblastoma cell lines (U87-MG, SNB-19, SF-268, and SF-295) were cultured with varying concentrations of COTI-2. A viable stain (alamarBlue) was added to cells after 4-7 days of drug exposure (approximately four doublings of control cells; maximum cell density in wells approximately 80%), and assayed for total cellular metabolic activity by absorbance. Concentrations of COTI-2 required to inhibit proliferation by 50% (IC_50_ value) were derived by interpolation of plotted data (mean values derived from three independent experiments ± SEM). **B.** U87-MG and SNB-19 glioblastoma cells were cultured overnight and then exposed to IC_50_ concentrations of COTI-2, cisplatin, or BNCU. Tumor cells were allowed to proliferate for 4-7 days then alamarBlue was used to measure the metabolic viability. Mean values obtained from three independent experiments ± SEM. *Significantly different from cells treated with cisplatin or BNCU using a Student's *t*-test (*p* < 0.05).

Adjuvant chemotherapy agents are among the most common regimens for the treatment of glioblastoma [13]. However, their effectiveness is limited and often accompanied by high toxicity [14]. Cisplatin and BCNU (carmustine) are often used against glioblastoma but patient response is limited [14]. We tested COTI-2 against multiple human glioblastoma tumors *in vitro*. As shown in Figure 3A, relatively low concentrations of COTI-2 were active against all human glioblastoma cell lines tested (U87-MG, SNB-19, SF-268, and SF-295). Two of the aforementioned cell lines, (U87-MG and SNB-19) were used to compare the efficacy of COTI-2, cisplatin, and BCNU. As shown in Figure 3B, COTI-2 showed superior activity and low IC_50_ when compared to cisplatin and BCNU in the treatment of human glioblastoma cell lines. These data collectively show that COTI-2 is more effective than some chemotherapy agents approved for treatment of glioblastoma.

### COTI-2 treatment induces apoptosis in cancer cells

**Figure 4:**
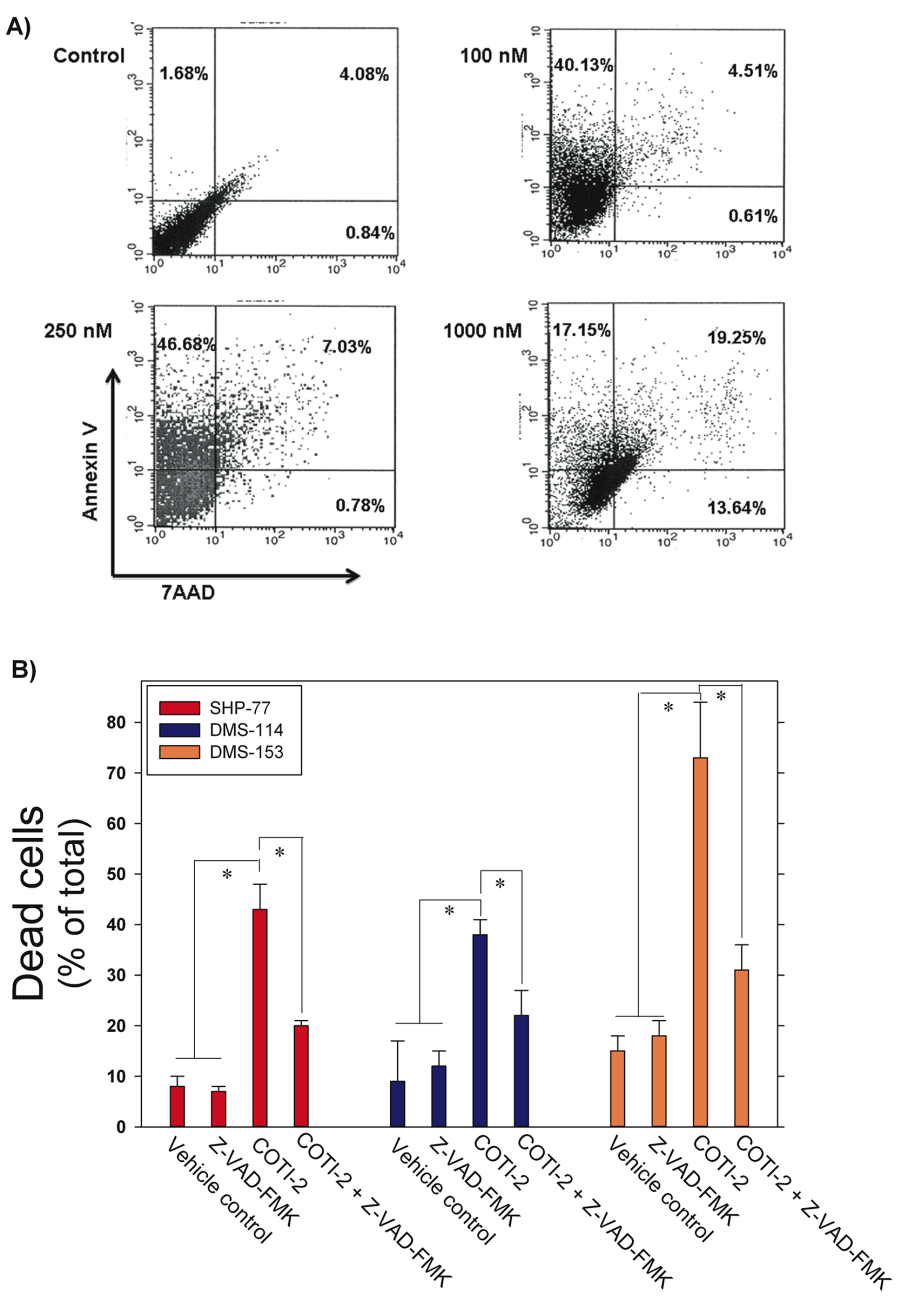
COTI-2 induces apoptosis in human cancer cells **A.** SHP-77 SCLC cells were treated with varying concentrations of COTI-2 for 48 h then stained with Annexin V and 7AAD to measure early and late apoptotic events. The lower left quadrant (Annexin V−/7AAD −) represents viable cells, while the upper left (Annexin V +/7AAD −) and upper right (Annexin V +/7AAD +) quadrants show apoptotic and necrotic cells/late apoptotic cells, respectively. **B.** Human SCLC cell lines (SHP-77, DMS-114, and DMS-153) were treated with the pan-caspase inhibitor Z-VAD-FMK for 1 h before culture media was replaced with fresh medium containing Z-VAD-FMK with or without COTI-2. Cells were cultured for 24 h then examined for apoptosis through Annexin V and PI staining. Cells were analyzed with flow cytometry. Mean values are obtained from three independent experiments ± SEM. *Significantly different from cells treated with vehicle, Z-VAD-FMK, or Z-VAD-FMK + COTI-2 using a Student's *t*-test (*p* < 0.05).

COTI-2 is effective against a wide variety of human tumor cells (Figure 1B). In an effort to understand the mechanism by which COTI-2 inhibited cell proliferation, we examined the capacity of COTI-2 to induce apoptosis in multiple human tumor cell lines. SHP-77 small cell lung cancer (SCLC) cells were exposed to various concentrations of COTI-2 for 48 h then stained with Annexin V and 7AAD to measure early and late apoptotic events [15]. COTI-2 treatment of SHP-77 cells with approximate IC_50_ concentrations resulted in the induction of early apoptosis among 40 to 47% of total cells (Figure 4A). COTI-2 treatment of SHP-77 cells with the IC_90_ concentration induced early and late apoptosis/necrosis in most cells. These data indicate that COTI-2 has cytotoxic effects against cancer cells even in nanomolar concentrations, primarily through induction of apoptosis.

To further elucidate the effect of COTI-2 on cancer cells, including induction of apoptosis, we treated three human SCLC cell lines (SHP-77, DMS-114, and DMS-153) with IC_50_ concentrations of COTI-2 following incubation with or without the pan-caspase inhibitor Z-VAD-FMK. This cell-permeable inhibitor prevents apoptosis induction in target cells by irreversibly binding to the catalytic site of caspase proteases [16]. Z-VAD-FMK-mediated inhibition of caspase cascades in all three SCLC cell lines to almost completely abrogate apoptosis, confirming the role of COTI-2 in inducing apoptosis in cancer cells via activation of the caspase cascade (Figure 4B).

Previous gene expression analysis studies suggested involvement of the PI3K/AKT/mTOR pathway in the mechanism of action of COTI-2 (our unpublished data). Activated AKT that is phosphorylated at Ser473 and Thr308 amino acid residues is thought to be responsible for the inactivation of caspase-9 [17, 18]. Unphosphorylated caspase-9 undergoes cleavage to active caspase-9 by an auto-proteolytic mechanism [18]. Phosphorylation of caspase-9 by phospho-Akt (p-AKT) impedes this process and thereby prevents downstream activation of caspase-3 [19]. We therefore examined the effect of COTI-2 on p-AKT and phosphorylated caspase-9 (p-caspase-9) in SHP-77 cells. As shown in Supplementary Figure S1, COTI-2 treatment reduced p-AKT and p-caspase-9 levels in SHP-77 cells, further establishing its role in inducing apoptosis via caspase pathways in cancer cells.

### COTI-2 effectively inhibits the growth of HT-29 and SHP-77 xenografts

**Figure 5:**
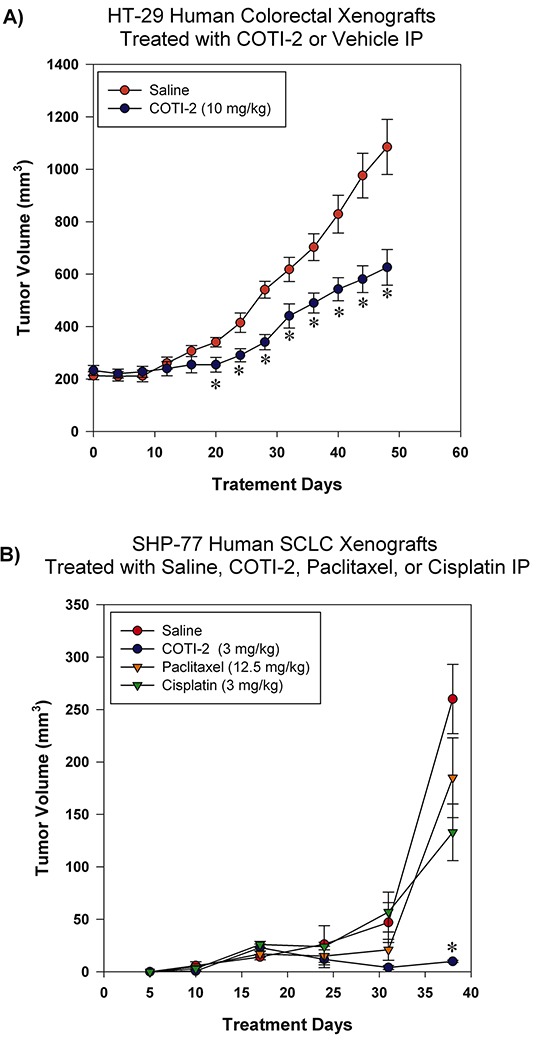
COTI-2 treatment inhibits human HT-29 and SHP-77 xenograft growth **A.** HT-29 human colorectal tumor cells (2 × 10^6^) were injected into the flanks of NCr-*nu* mice (n = 5 mice per group). Xenografts were allowed to reach ~ 200 mm^3^ before IP treatment initiation with COTI-2 (10 mg/kg, 5 days a week for 7 weeks) or saline alone. Tumor growth was measured every 4 days by caliper measurement. *Significant difference from saline treatment, Student's *t*-test, *p* < 0.05. **B.** Two million SHP-77 SCLC cells were injected into the flanks of NCr-*nu* mice (n = 5 mice per group). One day after injection of SHP-77 cells, animals received either 3 mg/kg of COTI-2 (once every two days, up to 38 days), 12.5 mg/kg of paclitaxel in 0.5 ml isotonic saline (once every two days, up to 38 days), or 3 mg/kg of cisplatin in isotonic saline (once per week for four weeks) IP. Tumor sizes were examined with caliper measurements. *Significant difference, Student's *t*-test, *p* < 0.05.

We assessed the effectiveness of COTI-2 in inhibiting the growth of HT-29 and SHP-77 xenografts in immunocompromised mice when administered intraperitoneally (IP). COTI-2 significantly inhibited tumor growth in the HT-29 human colorectal tumor xenografts at a dose of 10 mg/kg (Figure 5A). In addition to reducing tumor volumes at specific times post-treatment, COTI-2 also delayed the time required for tumors to reach specified volumes. For example, control tumors in mice treated with vehicle alone took only 32 days to reach a mean volume of 618 mm^3^, while tumors in mice treated with COTI-2 took 50% longer (48 days) to reach a similar mean volume (626 mm^3^)(Figure 5A). COTI-2 also significantly inhibited tumor growth in the SHP-77 SCLC xenograft model at a dose as low as 3 mg/kg. Calculation of COTI-2-induced delay in time to reach similar volume was not possible in the case of SHP-77 xenografts, as those tumors never reached average volumes similar to controls. In fact, COTI-2 was significantly more effective than cisplatin and paclitaxel at inhibiting SHP-77 xenograft growth (Figure 5B).

### COTI-2 treatment delays U87-MG and MDA-MB-231 xenograft growth

**Figure 6:**
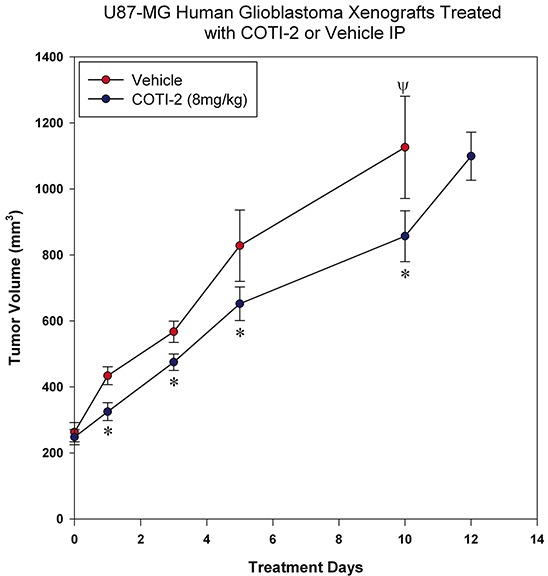
COTI-2 treatment delays U87-MG xenograft growth U87-MG glioblastoma cells (2 × 10^6^) were injected into the hind legs of the nude mice (7 mice per group). Tumors were allowed to grow to 200-300 mm^3^ before animals received COTI-2 treatment (8 mg/kg, 3 times per week) IP. *Significant difference, Student's *t*-test, *p* < 0.05. Ψ Indicates the day on which all animals in the control group were euthanized.

Glioblastoma is an aggressive form of brain cancer with low responsiveness to chemotherapy [14]. COTI-2 reduced tumor cell proliferation in multiple glioblastoma cell lines *in vitro* (Figure 1B). We therefore examined the ability of COTI-2 to reduce U87-MG xenograft growth *in vivo* when administered IP. U87-MG is a particularly aggressive glioblastoma model that is hard to control with chemotherapy [14]. Similar to the effect on HT-29 tumors, COTI-2 treatment both reduced U87-MG tumor volumes at specific times post-treatment and lengthened the time required for U87-MG xenografts to grow in nude mice (Figure 6). Control tumors in mice treated with vehicle alone took only 5 days to reach an average volume of 828 mm^3^ while tumors in animals treated with COTI-2 took double that time (10 days) to reach a similar mean volume (857 mm^3^). We further examined the capacity of COTI-2 to delay tumor growth in an aggressive breast cancer model (MDA-MB-231) when administered orally or *per os* (PO) in animals. PO treatment with COTI-2 delayed tumor xenograft growth (Supplementary Figure S2).

### COTI-2 treatment effectively inhibits OVCAR-3 xenograft growth

**Figure 7:**
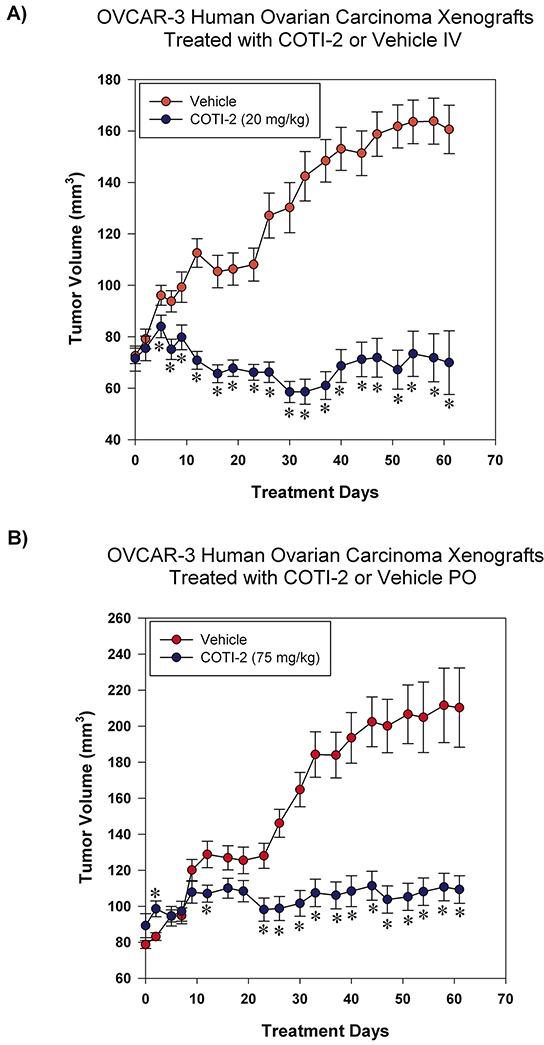
COTI-2 treatment inhibits OVCAR-3 xenograft growth OVCAR-3 ovarian carcinoma cells (7 × 10^6^) were injected SC over both thighs of NIH III *nu/nu* mice (n = 10-16 mice per treatment group). Tumors were allowed to grow to approximately 75-100 mm^3^ before the start of treatment. Mice received control vehicle (phosphate-citrate buffer without COTI-2, pH 3) or COTI-2 either **A.** IV (20 mg/kg, 3 times per week) or **B.** PO (75 mg/kg, 5 times per week). *Significant difference from control treatment, Student's *t*-test, *p* < 0.05.

COTI-2 was effective in inhibiting the proliferation of multiple ovarian tumor cell lines in the nanomolar range (Figure 1). We therefore examined the capacity of COTI-2 to inhibit OVCAR-3 tumor xenografts when treated intravenously (IV) or PO. As shown in Figure 7, COTI-2 treatment effectively inhibited OVCAR-3 xenograft growth regardless of the route of administration. In fact, significant inhibition was noted as early as the first week of treatment.

### COTI-2 treatment demonstrates a safe toxicity profile *in vivo*

**Figure 8:**
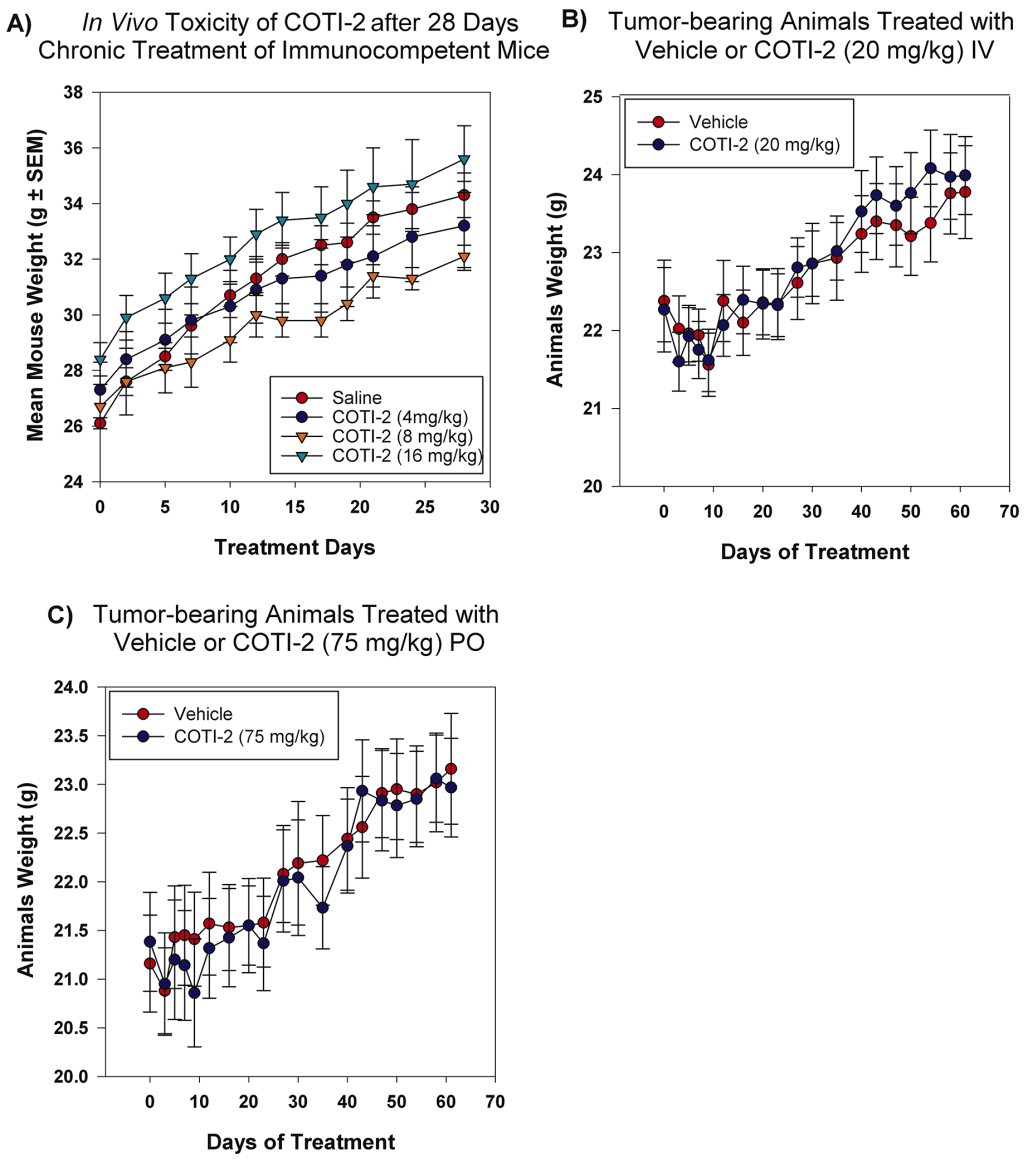
COTI-2 demonstrates a safe toxicity profile *in vivo* **A.** Immunocompetent mice (three per group) were treated with COTI-2 dosed IP in a 28-day chronic study. COTI-2 treatment did not result in mice losing weight or showing any sign of morbidity. **B.** Mice were transplanted with OVCAR-3 xenografts and tumors were allowed to grow to 100 mm^3^ before treatment with COTI-2. Animals received either vehicle or COTI-2 treatment IV (20 mg/kg, 3 times per week) or **C.** PO (75 mg/kg, 5 times per week) for 60 days before all the animals were sacrificed. COTI-2 treatment did not result in mice losing weight or showing any sign of morbidity.

Drug-induced toxicity is a major limiting factor in the success of all forms of cancer therapies, including cytotoxic chemotherapy, targeted-therapy, and most recently immunotherapy [20, 21]. Our CHEMSAS platform has the capacity to design molecules that have limited toxicity towards healthy cells and COTI-2 was designed for low toxicity against normal cells. We therefore examined the potential toxicity of COTI-2 in non-tumor-bearing mice over a 28-day chronic treatment study. As shown in Figure 8A, increasing doses of COTI-2 did not cause weight loss or any overt signs of morbidity in immunocompetent animals. Similar results were observed in the COTI-2 treatment of tumor-bearing mice (OVCAR-3 xenografts) where the mice tolerated the drug without displaying any signs of morbidity or weight loss (Figure 8B–8C). This suggests that COTI-2 selectively targets a wide variety of human cancer cell lines, as demonstrated by the *in vitro* data, while having little deleterious effects on normal cells.

## DISCUSSION

High-throughput screening (HTS) is widely used to identify new drug candidates. However, due to the intense resource consumption, low hit rates and unacceptably high failure rates in clinical trials, *in silico* drug design has gained popularity as a rational approach to predict clinical activity [22]. We have developed a proprietary, computer-based drug discovery technology (CHEMSAS) to identify a novel, small molecule, antineoplastic drug candidate called COTI-2. In the current study, we evaluated the *in vitro* and *in vivo* efficacy of COTI-2 in a variety of human cancers.

COTI-2 demonstrated effective anti-proliferative activity against solid epithelial cancer and leukemia cell lines (Figure 1B). Despite sharing common mutations, human cancers are strikingly heterogeneous, even among similar types of cancers, thus making them difficult to treat [23, 24]. We therefore assessed the efficacy of COTI-2 against multiple human cancer cell lines representing common human malignancies, allowing assessment of effectiveness of COTI-2 in cell lines with various mutations including; *TP53* (HT-29, HCT-15, OVCAR-3, K562, SF-268, SNB-19, T47D, MDA-MB-231), *KRAS* (MDA-MB-231, SW620), *PIK3CA* (MCF7, HT-29, T47D), *APC* (COLO-205, HCT-15), and *PTEN* (SF-295, SNB-19) [25]. COTI-2 appears to be a cytotoxic agent [26] since it inhibited cellular proliferation by inducing apoptosis in cancer cells (Figure 4). COTI-2-mediated cellular death was prevented when cancer cells were pre-treated with the pan-caspase inhibitor Z-VAD-FMK, confirming the activation of caspase cascades upon treatment with COTI-2 (Figure 4B).

Since COTI-2 was highly effective against a broad range of cancer types, our initial mechanistic studies involved the evaluation of COTI-2 as a kinase and Hsp90 ATPase inhibitor. To reduce the probability of false positives/negatives, COTI-2 was evaluated using two distinct kinase assays: an ATP-competitive binding assay and a radiometric functional assay. COTI-2 did not significantly inhibit over 200 kinases from major kinase pathways involved in cancer that were evaluated in both kinase assays (Supplementary Tables S2 and S3). Furthermore, COTI-2 did not inhibit the ATPase activity of Hsp90, a ubiquitous molecular chaperone that plays an essential role in cell survival and cell cycle control [27] (Supplementary Table S4). Ongoing studies suggest that COTI-2 may act primarily on mutated p53 and the PI3K/AKT/mTOR pathway (our unpublished data). Interestingly, most tested cell lines with mutant P53 were highly sensitive to COTI-2. However, there were other cell lines such as HCT-115, H292, and H460 with wild type P53 that were also highly sensitive to COTI-2 treatment. This suggests that COTI-2 may affect mutant P53 in human tumors, but likely has additional effects on other targets in the PI3K/AKT/mTOR pathway. COTI-2 also appears to negatively modulate the phosphorylation of AKT either directly or indirectly (our unpublished data). We confirmed that COTI-2 treatment reduced phosphorylated-AKT and -caspase-9 in SHP-77 cells suggesting a role for it negatively modulating the PI3K/AKT/mTOR pathway in cancer cells (Supplementary Figure S1). Further studies on the mechanism of action of COTI-2 are ongoing.

Despite important advances, many current chemotherapeutic regimens and targeted therapeutic drugs demonstrate poor efficacy [28, 29]. We decided to compare the efficacy of COTI-2 *in vitro* against standard chemotherapeutics and approved targeted-therapy agents such as cisplatin, BCNU, cetuximab, and erlotinib.

Cetuximab and erlotinib are targeted therapies against epidermal growth factor receptor (EGFR), a protein kinase with a crucial role in cell death and survival [30]. Erlotinib inhibits ATP-binding of the EGFR tyrosine kinase and is approved for treatment of metastatic NSCLC and advanced pancreatic cancer [11]. Additional indications, such as recurrent or metastatic colorectal cancer, are being explored in clinical trials (NCT00032110). We compared the efficacy of erlotinib to COTI-2 in NSCLC and colorectal cancer cell lines since erlotinib has been approved or is being investigated for the treatment of these indications (Figure 2 and Supplementary Table S1). COTI-2 was more effective at inhibiting the proliferation of both H292 and H1975 NSCLC cells when compared to erlotinib *in vitro*. As expected, erlotinib was more potent in the H292 than H1975 cells since the latter cell line carries a T790M EGFR mutation that confers resistance to erlotinib [31]. COTI-2, on the other hand, was equally effective in these cell lines regardless of the EGFR mutation. All the tested colorectal cancer cells line (SW620, COLO-205, and HCT-15) showed resistance to the tested doses of erlotinib but were sensitive to nanomolar concentrations of COTI-2 (Figure 2).

Cetuximab is approved for treatment of colorectal and metastatic head and neck cancer [8], and is currently being investigated as therapy for NSCLC (NCT00492206) and pancreatic cancer (NCT00305760) [32]. We compared its efficacy to COTI-2 in SW620, COLO-205, and HCT-15 colorectal cancer cell lines. All three were relatively resistant to cetuximab but sensitive to COTI-2 (Figure 2). This is significant because KRAS mutations mediate resistance to cetuximab and approximately 50% of colorectal cancer patients display this mutation [33]. Our data suggests that COTI-2 is effective against colorectal cancer cell lines regardless of their KRAS mutation status.

Current treatment strategy for malignant glioblastoma multiforme [34], an aggressive adult and pediatric brain cancer, includes agents with limited activity such as temozolomide, cisplatin, and BCNU [14]. The DNA damage-inducing agents cisplatin and BCNU [35, 36] were not as effective as COTI-2 in reducing proliferation of U87-MG and SNB-19 glioblastoma cells *in vitro* (Figure 3). In fact, COTI-2 was 10 to 500 times more effective than these agents, suggesting that COTI-2 is more efficacious than standard chemotherapeutic agents when tested against human glioblastoma cell lines.

We investigated the *in vivo* efficacy of COTI-2 in 5 tumor xenograft models in mice (HT-29 colorectal cancer, SHP-77 SCLC, U87-MG glioblastoma, MDA-MB-231 breast cancer, and OVCAR-3 ovarian cancer). The xenograft tumors were established by subcutaneous inoculation of tumor cells into nude mice and COTI-2 was administered either IP, PO, or IV. COTI-2 induced significant tumor growth delay in both the particularly aggressive [37, 38] HT-29 (Figure 5A) and U87-MG (Figure 6A) xenograft models when administered IP. When compared to the standard chemotherapeutics cisplatin and paclitaxel [39], COTI-2 significantly delayed the growth of SHP-77 xenografts in immunocompromised mice when administered IP (Figure 5B). Depending on the xenograft model, tumor volumes after treatment with COTI-2 were as low as 5% of the volumes of control tumors in mice treated with vehicle alone.

COTI-2 was also equally effective when the route of administration was changed from IP to PO in both the MDA-MB-231 and OVCAR-3 tumor xenografts. Following PO treatment, COTI-2 significantly reduced tumor growth in the MDA-MB-231 breast cancer xenografts (Supplementary Figure S2). COTI-2 was most effective against human ovarian carcinoma OVCAR-3 xenografts whether administered IV or PO (Figure 7). In fact, tumor regression was observed following IV administration of COTI-2 in this model (Figure 7A) while tumor volumes remained relatively low and stable over the 60 days of PO treatment (Figure 7B). COTI-2 administered repeatedly for 28 days (monitored for up to 60 days) did not induce overt signs of toxicity or weight loss, whether administered IV, PO, or IP, or to tumor-bearing immunocompromised mice or immune-competent mice (Figure 8).

Overall, our data shows that COTI-2 is a novel small molecule active against a wide variety of human tumor cell lines and xenografts that are traditionally difficult to treat. It activates the caspase signaling cascade, thereby apoptosis in tumor cells. COTI-2 also shows enhanced activity compared to some first-line chemotherapy drugs and targeted-therapy agents. It is active regardless of the route of administration. Most importantly, COTI-2 is well-tolerated *in vivo* and shows a favorable safety profile in mice with different genetic backgrounds. Although the precise mechanism of action of COTI-2 is still under investigation, the data indicates that it is not a traditional kinase or Hsp90 inhibitor. Additional studies to further elucidate the mechanism of action of COTI-2 are currently underway.

## MATERIALS AND METHODS

### Cell culture

Authenticated human small cell lung cancer (SCLC) (DMS-114, DMS-153, and SHP-77), non-small cell lung cancer (NSCLC) (A549, H226, H292, H1975, and NCI-H460), human colorectal cancer (SW620, COLO-205, HCT-15, HCT-116, and HT-29), human glioma/astrocytoma (SF-268, SF-295, SNB-19, and U87-MG), human ovarian carcinoma (OVCAR-3, OVCAR-5, OVCAR-8 and CaOv-3), human breast cancer (MCF-7, T47D, and MDA-MB-231), human leukemia (HL60 and K562), human endometrial cancer (Ishikawa, Hec-1A, KLE, and RL-95-2), and human pancreatic cancer cell lines (PANC-1, Panc 02.03, Panc 03.27, Panc 10.05, Capan-2, BxPC-3, and AsPC-1) were obtained from American Type Culture Collection (ATCC), National Institute of Health (NCI) repository, or Charles River Laboratories amplified and frozen at −80°C and never passaged (upon thawing) for more than one month before use in an experiment. Cell lines were maintained under standard condition in 37°C in 5% CO_2_. All cell lines were tested to ensure viability and lack of contaminating mycoplasma and common viruses (Idexx Bioresearch IMPACT II RADIL testing), and their identity verified using the GenePrint® 10 System (10 markers) at The Centre for Applied Genomics, The Hospital for Sick Children, Toronto, Canada. All human tumor cell lines were seeded in tissue culture medium plus 10% fetal bovine serum (FBS, Invitrogen, Carlsbad, CA) at a cell density that allowed for growth to no more than 75-80% confluence by 3 or 4 days after seeding, depending on the experiment. OVCAR-3, OVCAR-5, OVCAR-8, AsPC-1, and BxPC-3 were grown in RPMI 1640 medium. Capan-2 and Hec1A were grown in McCoy's 5A medium. KLE cells were grown in DMEM-F12 medium. RL-95-2 cells were grown in DMEM-F12 + insulin (5 μg/ml). CaOV-3 and PANC-1 cells were grown in DMEM, and Ishikawa cells were grown in MEMα + NEAA (non-essential amino acids). All were supplemented with 10% FBS and cultured in a humidified 5% CO_2_ incubator without antibiotics. All other cell lines were cultured in MEMα + 10% FBS without antibiotics.

### Cytotoxic drugs

COTI-2 was supplied by Critical Outcome Technologies Inc. (London, Ontario). COTI-2 was dissolved in 100% dimethyl sulfoxide stock solution and diluted in medium plus FBS such that final DMSO concentrations were 0.5-1.0% depending on the experiment. Cetuximab was obtained from ImClone Systems (New York, NY). Erlotinib was purchased from LC Laboratories (Woburn, MA). Cisplatin (Sigma-Aldrich, St. Louis, MO), paclitaxel (Bristol-Myers Squibb Co. New York, NY), and BCNU (Carmustine) were obtained from the London Health Sciences Centre pharmacy (London, Ontario).

### Growth inhibition/cell viability assay

#### Proliferation assay (cell counting)

Tumor cell proliferation after drug treatment was described previously [40]. Briefly, tumor cells were washed with 1X PBS, trypsinized, and counted using a Beckman Coulter Z1 Particle Counter (Beckman, Mississauga, Ontario) three days after treatment at a cell density of no more than 80%. The fold-change in cell number after 72 h of growth (relative to the starting number of seeded cells) was calculated according to the following formula:

$$\text{Fold Change}=\frac{\left[\left(\text{Number of Cells, Day}3\right)-\left(\text{Number of Cells, Day}0\right)\right]}{\left(\text{Number of Cells, Day}0\right)}$$

The drug effects on proliferation were expressed as “Proliferation (% control)” and were calculated as follows:

$$\text{Proliferation }(\%\text{ Control})=\frac{\text{Treatment Fold Change}}{\text{Control Fold Change}}\times 100$$

The control cultures were treated with vehicle minus the drug. For all IC_50_ determinations, preliminary experiments were performed to determine the range of appropriate concentrations. A second and final series of experiments were then performed (4 independent experiments, n=3 replicate measures for each experiment) using COTI-2 or other drug concentrations higher and lower than the estimated IC_50_, and including concentrations that reduced proliferation by approximately 40% and 60%. The IC_50_ for each of the 4 experiments was determined by interpolation between these values, and error estimates (SEM) for the final IC_50_ calculated using the 4 values.

#### Proliferation assay (by alamarBlue^®^ measurement of metabolic activity)

Tumor cell proliferation was also measured by alamarBlue^®^ assay (Invitrogen, Carlsbad, CA). Viable tumor cells were plated in 96-well culture plates and allowed to adhere for 16 h, followed by addition of COTI-2 (in medium plus 10% FBS) at multiple concentrations ranging from those that had no effect on proliferation to those that inhibited proliferation by 90% or more. AlamarBlue was then added to the cells after 4-7 days of drug exposure (approximately 4 doublings of the control cells), and assayed for total cellular metabolic activity (a function of population density of live cells) by absorbance using a Wallac Victor2 multilabel counter (PerkinElmer Wallac, Gaithersburg, MD). Concentrations of the agent required to inhibit proliferation by 50% (IC_50_ value) compared to control cells treated with 0.5% DMSO in medium plus 10% FBS were derived by interpolation of plotted data (mean values derived from 3 independent experiments ± SD).

### Cell viability assay

Cell viability was measured by the CellTiter-Blue^®^ cell viability assay Promega (Madison, WI). This procedure measures the conversion of the indicator dye resazurin to resorufin in metabolically active cells, hence is an indicator of cell viability. Following treatment, growth media was removed and cells were incubated with 20 ml of CellTiter-Blue reagent and growth media for 1-4 h at 37°C. Fluorescence values were measured at 535/590 nm using a Beckman-Coulter DTX-880 microplate reader (Brea, CA).

### Analysis of apoptosis by flow cytometry

SHP-77 cells were cultured with various concentrations of COTI-2 for 48 h. Cells were then washed twice with 1X cold PBS and stained with Annexin V and 7AAD (BD Pharmingen, San Diego, CA) according to the manufacturer's instructions. Briefly, 5 μl of Annexin V and 7AAD were added to 1 × 10^5^ cells and incubated for 15 min at room temperature in the dark. Then 400 μl of the 1X binding buffer (100 mM HEPES, pH 7.4, 140 mM NaCl, 2.5 mM CaCl_2_) was added to the cells. Finally, cells were analyzed using a BD FACS Calibur flow cytometer (BD Biosciences, Franklin Lakes, NJ) and FlowJo software (Tree Star, Inc., Ashland, OR, USA).

### Immunoblot analysis of phosphorylation of AKT, STAT3, caspase-9, and cleavage of caspase-9

Cell extracts were prepared from SHP-77 cells and treated with COTI-2 (250 nM) or an equivalent volume of the DMSO suspension vehicle. Cells were incubated for 3 or 6 h, washed twice with ice-cold 1X PBS, harvested, and sonicated. Cell lysates were then centrifuged at 15000X RPM for 15 min at 4°C and the supernatant collected and stored at −80°C. A Bradford protein assay (BioRad, Hercules, CA) was used to measure protein concentrations. The proteins were then separated by electrophoresis and transferred to a PVDF membrane (Millipore Corp., Bedford, MA). The membranes were incubated with various primary antibodies, and then secondary antibody conjugated with peroxidase. The signal was visualized using a Storm scanner (GE Healthcare Life Sciences). The following antibodies, all from Cell Signaling Technology Inc. (Danvers, MA), were used to detect the proteins on the membrane: anti-Akt2 (#2962), anti-phospho-Akt (Ser473, #587F11), anti-STAT3 (#9132), anti-phospho-STAT3 (Tyr705, #9138S), anti-caspase-8 (1C12), anti-cleaved caspase-9 (Asp330, #9501), anti-phospho caspase-9 (Thr125, #2226S), and anti-β tubulin (#2128).

### Apoptosis assay

DMS-114, DMS-153, and SHP-77 human SCLC cells (1 × 10^5^) were cultured for 24 h before the medium was replaced with 1 ml of fresh medium containing the pan-caspase inhibitor Z-VAD-FMK (20 μM) (Sigma-Aldrich, St. Louis, MO). After 1 h the Z-VAD-FMK medium was removed and an equal volume of 2X medium (with Z-VAD-FMK, 20 μM) with or without COTI-2 was added to the cells at the IC_50_ concentrations for each cell line as follow: DMS-114 (51 nM), DMS-153 (73 nM), and SHP-77 (156 nM). Control cells that did not receive COTI-2 received 2X medium (with or without Z-VAD-FMK, 20 μM) only. The final solutions contained COTI-2 (at IC_50_), Z-VAD-FMK (0 or 10 μM), and RPMI-1640 + 10% FBS. After 24 h the cells were analyzed for apoptosis as follows: Supernatant medium containing non-adherent cells was collected. Adherent cells were rinsed with ice-cold PBS, trypsinized, and added to the non-adherent fraction. Cells were then washed twice in ice-cold PBS, re-precipitated by centrifugation, re-suspended (1000 cells/μl) in binding buffer (140 mM NaCl_2_/2.5 mM CaCl_2_/10 mM HEPES) and a fraction (100 μl) incubated in the dark with 10 μl propidium iodide (PI, 50 ng/ml, Sigma, St. Louis, MO) plus 2 μl Annexin V-FITC (25 ng/ml; BD Biosciences, Mississauga, Ontario). Samples were analyzed for combined Annexin V-FITC/PI staining (to detect late apoptotic cells and post-apoptotic cells) using an EPICS XL-MCL flow cytometer (Beckman Coulter, Hileah, FL). Data was analyzed using CellQuest software.

### ATP-competitive KINOMEscan^TM^ kinase assay

The interaction of COTI-2 with 227 kinases was tested using the AMBIT BIOSCIENCES KINOMESCAN assay (DiscoveRx Corp., Silver Spring, MD) as described previously [41]. In brief, streptavidin-coated magnetic beads were treated with biotinylated small molecule ligands for 30 min at 25°C to generate affinity resins for kinase assays. The liganded beads were blocked with excess biotin and washed with blocking buffer (1% BSA, 0.05% Tween 20, 1 mM DTT) to remove unbound ligand and to reduce non-specific binding. Binding reactions were assembled by combining phage lysates, liganded affinity beads, and COTI-2 in 1X binding buffer (20% SeaBlock, 0.17X PBS, 0.05% Tween 20, 6 mM DTT). COTI-2 was prepared as a 1,000X stock in DMSO and rapidly diluted into the aqueous environment (0.1% DMSO final). DMSO (0.1%) was added to control assays lacking the test compound. All reactions were carried out in polystyrene 96-well plates that had been pre-treated with blocking buffer in a final volume of 0.1 ml. The assay plates were incubated at 25°C with shaking for 1 h, long enough for binding reactions to reach equilibrium, and the affinity beads were washed 4X with wash buffer (1X PBS, 0.05% Tween 20, 1mM DTT). After the final wash, the beads were re-suspended in elution buffer (1X PBS, 0.05% Tween 20, 2 μM non-biotinylated affinity ligand) and incubated at 25°C with shaking for 30 min. The eluates were measured by standard plaque assays or by quantitative PCR. The binding constant (Kd) was determined as described previously [42].

The % control calculation is as follows:

$$\%\text{ Control }=\frac{\text{Test Com pound Signal}-\text{ Positive Control Signal}}{\text{Negative Control Signal}-\text{ Positive Control Signal}}\times 100$$

### Radiometric functional kinase assay

In the radiometric based filtration binding assay, the kinase reaction is performed in the presence radioisotope labelled γ-ATP which is incorporated into the kinase substrate that is then detected after washing away unreacted phosphate [43]. The advantage of this method is that it can be used for any kinase and substrate without limitation since it does not rely on special substrate labeling or modification and detection is free from interference. Each substrate was prepared in fresh reaction buffer (20 mM Hepes [pH 7.5], 10 mM MgCl_2_, 1 mM EDTA, 0.02% Brij35, 0.02 mg/ml BSA, 0.1 mM Na_3_V0_4_, 2 mM DTT, and 1% DMSO). Required cofactors and test kinases were each added to the aforementioned substrate solution and mixed gently. The target compounds in 100% DMSO were diluted to the appropriate concentration into the kinase reaction mixture using Acoustic Technology (Echo550; nanoliter range). Then ^33^P-γ-ATP was added to initiate the reaction and incubated for 2 h at room temperature. The kinase activity was detected by P81 filter-binding method (http://reactionbiology.com/webapps/site/Kinase_Assay_Protocol.aspx).

### Inhibition of Hsp90 ATPase activity

The ability of test compounds to compete with a fluorescently labelled probe for binding to full-length recombinant human Hsp90 was determined by means of a suited fluorescence polarization assay, as previously described [43]. The assay is based on the competition of fluorescently-labeled geldanamycin (FITC-GM) for binding to Hsp90. FITC-GM binds to the ATP-binding pocket of Hsp90, therefore, ATP-competitive inhibitors can be identified using this assay. COTI-2 was tested in a 10-dose IC_50_ in duplicate with 3-fold serial dilution starting at 100 μM against Hsp90α and Hsp90β. Control compounds radicicol and geldanamycin were tested in a 10-dose IC_50_ with 3-fold serial dilution starting at 10 μM.

### *In vivo* xenograft studies

SHP-77 and HT-29 cells were injected in 50% matrigel (ECM gel, Sigma-Aldrich, St. Louis, MO) into flanks of NCr-*nu* mice (2 × 10^6^ cells per injection site) (n= 5 mice per group). In the case of SHP-77 xenografts, treatment with COTI-2 began prior to the appearance of palpable tumors (i.e., the capacity of COTI-2 to suppress growth of nascent tumors was assessed). One day after injection of SHP-77 cells, animals received either 3 mg/kg of COTI-2 (once every two days, up to 38 days), 12.5 mg/kg of paclitaxel in 0.5 ml isotonic saline (once every two days, up to 38 days) [44], or 3 mg/kg of cisplatin in isotonic saline (once per week for four weeks) IP [45]. Tumor sizes were estimated at 5, 10, 17, 24, and 38 days, by standard caliper measurements. In the case of HT-29 xenografts, the capacity of COTI-2 to suppress growth of established tumors was assessed. HT-29 xenografts were allowed to grow to 200 mm^3^ before starting IP treatment with COTI-2 (10 mg/kg, 5 days a week for 7 weeks) or saline IP. Tumor growth was measured every 4 days by caliper measurement. U87-MG cells (2 × 10^6^) were injected in 50% matrigel subcutaneously (SC) into the hind legs of nude mice (2 injection sites per animal). Treatment with COTI-2 (8 mg/kg in isotonic saline) started when U87-MG xenografts reached 200-300 mm^3^. Control mice were treated with isotonic saline alone. Animals were treated 3 times per week IP and tumor growth was measured with caliper. MDA-MB-231 breast tumor cells (2 × 10^6^) were injected into the flanks of 6-8 week old SCID mice. Animals were divided into two groups (n=7 mice/group) as follows: Group 1 – oral gavage with phosphate-citrate buffer alone (pH 2.3), 5 days per week, 100 μl per gavage, until tumors reached a maximum of 1 cm^3^; Group 2– oral gavage with COTI-2 (200 mg/kg in phosphate-citrate buffer, pH 2.3), 5 days per week, 100 μl per gavage, commencing the day the mean tumour volume in this group reached 100-200 mm^3^. Tumor volumes in each group were assessed by caliper measurement. OVCAR-3 human ovarian carcinoma cells (7 × 10^6^) were injected SC over both thighs of 4-8 weeks old female NIH III *nu/nu* mice (n = 10-16 mice per treatment group). When tumors reached a palpable volume of approximately 75-100 mm^3^, treatment with control vehicle (phosphate-citrate buffer without COTI-2, pH 3) or with vehicle plus COTI-2 was initiated either IV (20 mg/kg, 3 times per week) or PO (75 mg/kg, 5 times per week). Treatments were continued until tumors in mice treated with control vehicle reached the maximum volume allowable according to the animal care guidelines described by the Canadian Council on Animal Care and monitored by The Western University Animal Use Subcommittee, or maximum permissible measures and markers of discomfort were reached (*i.e.,* mouse discomfort or body weight loss reached maximum allowable levels). Tumor volumes were examined using caliper measurements.

All tumor volumes in this study were estimated from caliper measurements of length and width then calculated as follow: π/6 × (longest diameter) × (shortest diameter)^2^. Animal handling and procedures were conducted according to the animal experimentation guide and protocols of the Animal Use Subcommittee of Western University.

### *In vivo* toxicity studies

Adult BALB/c mice (8 weeks old) were injected IP with COTI-2 dissolved in saline at 4, 8, and 16 mg/kg three times per week for 28 days (three mice per group). Animals were weighed prior to each injection at each of the indicated days (0, 2, 5, 7, 10, 12, 14, 17, 19, 21, 24, and 28). Subjective indicators of toxicity such as bruising at the injection site, alopecia, and immobility were monitored for a 30-min period following dosing and 24 h post-dosing. Similar measures of toxicity were also evaluated in the mice tested for the OVCAR-3 tumor xenograft model.

### Statistical analysis

Student's *t* test (2-tailed) was used to determine differences between two means. One-way ANOVA was used to assess differences among multiple means. A *p* value of 0.05 was selected *a priori* to indicate significant differences.

## SUPPLEMENTARY FIGURES AND TABLES






