# Correction: COTI-2, a novel small molecule that is active against multiple human cancer cell lines *in vitro* and *in vivo*

**DOI:** 10.18632/oncotarget.20600

**Published:** 2017-09-01

**Authors:** Kowthar Y. Salim, Saman Maleki Vareki, Wayne R. Danter, Serban San-Marina, James Koropatnick

**Affiliations:** ^1^ Critical Outcome Technologies Inc., London, Ontario, Canada; ^2^ Cancer Research Laboratory Program, Lawson Health Research Institute, London, Ontario, Canada; ^3^ Department of Microbiology and Immunology, Western University, London, Ontario, Canada; ^4^ Department of Pathology, Western University, London, Ontario, Canada; ^5^ Department of Oncology, Western University, London, Ontario, Canada; ^6^ Department of Physiology and Pharmacology, Western University, London, Ontario, Canada; ^7^ Department of Otolaryngology-Head and Neck Surgery, Mayo Clinic School of Medicine, Rochester, Minnesota, USA

**This article has been corrected:** Dr. Serban San-Maria was added to the author list.

The authors sincerely apologize for this oversight.

Original article: Oncotarget. 2016; 7:41363-41379. https://doi.org/10.18632/oncotarget.9133

