# Supplementary material for: COTI-2, a novel small molecule that is active against multiple human cancer cell lines in vitro and in vivo
Source: Oncotarget. 2016 May 2;7(27):41363–79. doi: 10.18632/oncotarget.9133 (PMC5173065; doi:10.18632/oncotarget.9133)
Supplement: Supplementary file 1 [file oncotarget-07-41363-s001.pdf]

SUPPLEMENTARY FIGURES AND TABLES

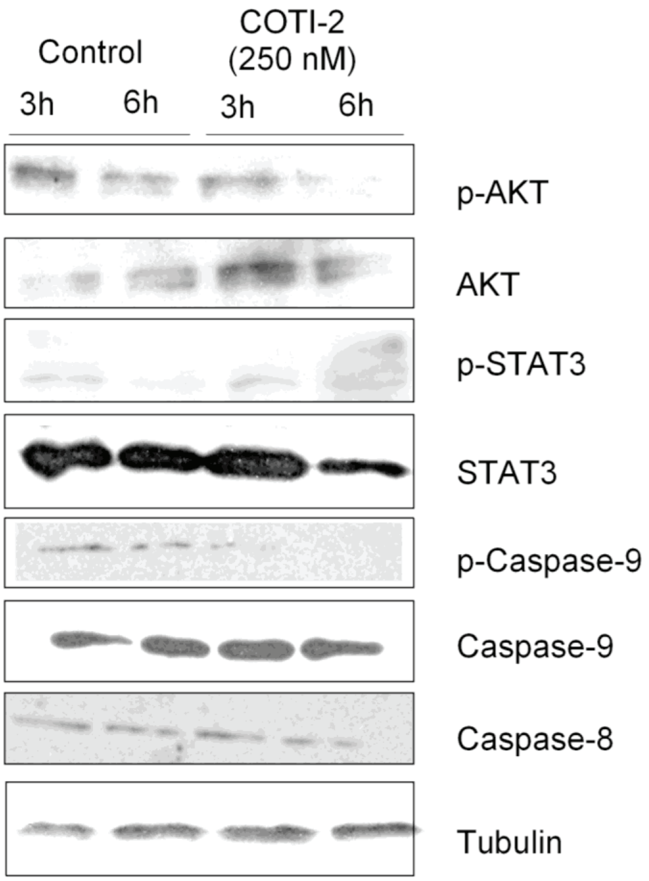

Supplementary Figure S1: COTI-2 treatment reduces p-AKT and p-caspase-9 levels in SHP-77 cells. p-AKT, p-STAT3, and p-Caspase-9 levels were measured in cell extracts of SHP-77 cells treated with COTI-2 (250 nM) or vehicle. Tumor cells were incubated with COTI-2 for 3 or 6 h before cells were lysed and proteins were extracted. COTI-2 treatment reduced p-AKT and p-caspase-9 levels in the treated cells.

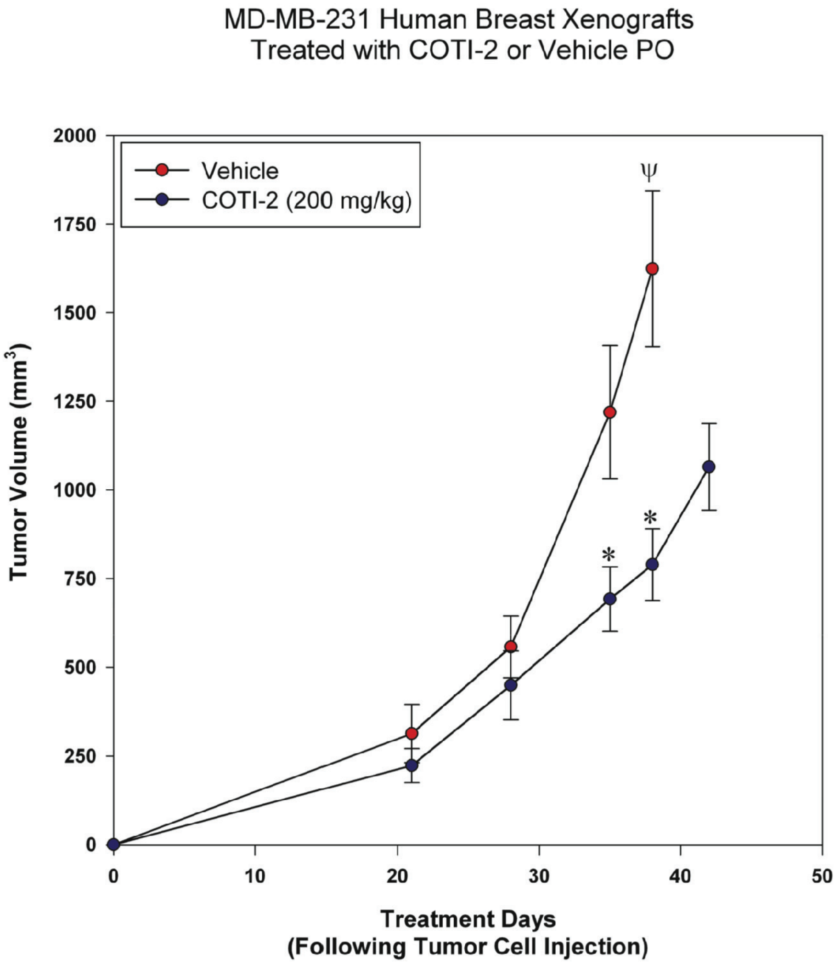

Supplementary Figure S2: Two million MDA-MB-231 breast tumor cells were injected into the flanks of SCID mice (7 mice per group). Xenografts were allowed to grow to 100-200 mm^3 before animals were dosed with COTI-2 (200 mg/kg in phosphate-citrate buffer, pH 2.3) at 5 days per week, 100 µl per PO gavage until tumors reached 1 cm^3. *Significant difference from control treatment, Student's *t*-test, $p < 0.05$. Ψ Indicates the day on which all animals in the control group were euthanized.

Supplementary Table S1: COTI-2 treatment is more effective than erlotinib in controlling NSCLC cell line growth. H292 and H1975 NSCLC cells were cultured in the presence of varying concentrations of COTI-2 or erlotinib for 72 h. Cell viability was then measured by the CellTiter-Blue® cell viability assay. Each of the data points is an average of two measurements

COTI-2 (µM)	H1975 cell proliferation (% of control)	H292 cell proliferation (% of control)	Erlotinib (µM)	H1975 cell proliferation (% of control)	H292 cell proliferation (% of control)
0.01	105	42	0.01	94	61
0.1	63	35	0.1	108	50
1	10	40	1	92	46
10	4	0.3	10	76	26

Supplementary Table S2: COTI-2 is not a kinase inhibitor as determined by a radiometric functional kinase assay. COTI-2 was tested in a 10-dose IC_{50} mode with 3-fold serial dilution starting at 1 or 10 µM. Control compounds included either staurosporine or LY294002 were tested in a 10-dose IC_{50} mode with 5-fold or 3-fold serial dilution starting at 20 or 100 µM. Reactions were carried out at 1 or 10 µM ATP. ND indicates that the compound was not tested against the kinase. The IC_{50} of the following kinases could not be determined because the shape of the dose-response curve is not interpretable: CAMK1d, CDK9/cyclinT1, CTK/MATK/HYL, FGFR4, PKCd.

See Supplementary File 1

Supplementary Table S3: COTI-2 is not a traditional kinase inhibitor as determined by an ATP-competitive KINOMEscan™ kinase assay. COTI-2 was screened against a panel of kinases at a single concentration of 10 µM and the quantitative dissociation constant (K_d) was determined. The dissociation constant (K_d) is a measure of binding specificity such that $K_d > 1$ µM is considered relatively weak binding.

See Supplementary File 2

Supplementary Table S4: COTI-2 does not inhibit Hsp90 ATPase activity. The assay is based on the competition of fluorescently labeled geldanamycin (FITC-GM) for binding to Hsp90. COTI-2 was tested in a 10-dose IC_{50} in duplicate with 3-fold serial dilution starting at 100 µM. Control compounds, geldanamycin and radicicol, were tested in a 10-dose IC_{50} with 3-fold serial dilution starting at 10 µM

Compound ID	Compound IC_{50} to HSP90α	Compound IC_{50} to HSP90β
COTI-2	No inhibition	No inhibition
Geldanamycin	2.18×10^{-8}	2.49×10^{-8}
Radicicol	3.88×10^{-8}	7.30×10^{-7}
