# Supplementary material for: COTI-2, a novel small molecule that is active against multiple human cancer cell lines in vitro and in vivo
Source: Oncotarget. 2016 May 2;7(27):41363–79. doi: 10.18632/oncotarget.9133 (PMC5173065; doi:10.18632/oncotarget.9133)
Supplement: Supplementary file 2 [file oncotarget-07-41363-s002.docx]

**Supplementary Table 2**. COTI-2 is not a kinase inhibitor as determined by a radiometric functional kinase assay. COTI-2 was tested in a 10-dose IC_50_ mode with 3-fold serial dilution starting at 1 or 10 µM. Control compounds included either staurosporine or LY294002 were tested in a 10-dose IC_50_ mode with 5-fold or 3-fold serial dilution starting at 20 or 100 µM. Reactions were carried out at 1 or 10 µM ATP. ND indicates that the compound was not tested against the kinase. The IC_50_ of the following kinases could not be determined because the shape of the dose-response curve is not interpretable: CAMK1d, CDK9/cyclinT1, CTK/MATK/HYL, FGFR4, PKCd.

| **Kinase** | **COTI-2**  **IC_50_ (nM)** | **Staurosporine**  **IC_50_ (nM)** | **LY294002**  **IC_50_ (nM)** |
| --- | --- | --- | --- |
| ABL1 | No inhibition | 45.17 | ND |
| ABL2/ARG | No inhibition | 11.60 | ND |
| AKT1 (dPH, S473D) | No inhibition | 2.81 | ND |
| AKT2/PKBb | No inhibition | 3.13 | ND |
| AKT3 | No inhibition | 1.65 | ND |
| ALK | No inhibition | 2.84 | ND |
| ALK4/ACVR1B | No inhibition | 2122.00 | ND |
| ALK5/TGFB-R1 | No inhibition | 7998.00 | ND |
| ARK5 | No inhibition | 0.52 | ND |
| ASK1/MAP3K5 | No inhibition | 24.39 | ND |
| Aurora A | No inhibition | 1.12 | ND |
| Aurora B | No inhibition | 6.58 | ND |
| Aurora C /AURKC | No inhibition | 2.26 | ND |
| AXL | No inhibition | 20.10 | ND |
| BLK | No inhibition | 2.65 | ND |
| BMX | No inhibition | 7.91 | ND |
| BRAF | No inhibition | No inhibition | ND |
| BRK | No inhibition | 62.06 | ND |
| BRSK1 | No inhibition | 0.16 | ND |
| BRSK2 | No inhibition | 7.04 | ND |
| BTK | No inhibition | 21.17 | ND |
| c-Kit | No inhibition | 35.77 | ND |
| c-MER | No inhibition | 11.29 | ND |
| c-MET | No inhibition | 67.46 | ND |
| c-Src | No inhibition | 1.87 | ND |
| CAMK1a | No inhibition | 6.26 | ND |
| CAMK2a | No inhibition | 0.27 | ND |
| CAMK4 | No inhibition | 92.19 | ND |
| CAMKIIb | No inhibition | 0.03 | ND |
| CAMKIIdelta | No inhibition | 0.05 | ND |
| CAMKIIgamma | No inhibition | 0.08 | ND |
| CAMKK1 | No inhibition | 44.69 | ND |
| CAMKK2 | No inhibition | 35.79 | ND |
| CDK1/cyclinB | No inhibition | 1.37 | ND |
| CDK2/cyclinA | No inhibition | 0.62 | ND |
| CDK2/cyclinE | No inhibition | 3.75 | ND |
| CDK3/cyclinE | No inhibition | 6.23 | ND |
| CDK5/p25 | No inhibition | 4.55 | ND |
| CDK5/p35 | No inhibition | 1.59 | ND |
| CDK6/cyclinD1 | No inhibition | 8.07 | ND |
| CDK7/cyclinH/MNAT1 | No inhibition | 4301.00 | ND |
| CHK1 | No inhibition | 0.23 | ND |
| CHK2 | No inhibition | 5.38 | ND |
| CK1a1 | No inhibition | 2159.00 | ND |
| CK1d | No inhibition | 7251.00 | ND |
| CK1epsilon | No inhibition | 904.30 | ND |
| CK1g1/CSNK1G1 | No inhibition | 11260.00 | ND |
| CK1g2 | No inhibition | 2041.00 | ND |
| CK1g3/CSNK1G3 | No inhibition | 2256.00 | ND |
| CK2a | No inhibition | 4528.00 | ND |
| CK2a2 | No inhibition | 1621.00 | ND |
| CLK1 | No inhibition | 31.19 | ND |
| CLK2 | No inhibition | 33.86 | ND |
| CLK3 | No inhibition | 2273.00 | ND |
| CLK4 | No inhibition | 38.74 | ND |
| COT1/MAP3K8 | No inhibition | 31.52 | ND |
| CSK | No inhibition | 32.83 | ND |
| DAPK1 | No inhibition | 5.33 | ND |
| DAPK2 | No inhibition | 5.48 | ND |
| DCAMKL2 | No inhibition | 54.30 | ND |
| DDR2 | No inhibition | 1.00 | ND |
| DMPK | No inhibition | 38.01 | ND |
| DNA-PK | No inhibition | ND | 90.05 |
| DRAK1/STK17A | No inhibition | 4.54 | ND |
| DYRK1B | No inhibition | 7.11 | ND |
| DYRK1/DYRK1A | No inhibition | 32.00 | ND |
| DYRK2 | No inhibition | 375.40 | ND |
| DYRK3 | No inhibition | 287.70 | ND |
| DYRK4 | No inhibition | >20000 | ND |
| EGFR | No inhibition | 19.07 | ND |
| EPHA1 | No inhibition | 39.70 | ND |
| EPHA2 | No inhibition | 80.36 | ND |
| EPHA3 | No inhibition | 29.56 | ND |
| EPHA4 | No inhibition | 11.73 | ND |
| EPHA5 | No inhibition | 12.57 | ND |
| EPHA7 | No inhibition | 29.45 | ND |
| EPHA8 | No inhibition | 94.63 | ND |
| EPHB1 | No inhibition | 20.68 | ND |
| EPHB2 | No inhibition | 70.21 | ND |
| EPHB3 | No inhibition | 1681.00 | ND |
| EPHB4 | No inhibition | 359.60 | ND |
| ErbB2/HER2 | No inhibition | 511.00 | ND |
| ErbB4/HER4 | No inhibition | 333.50 | ND |
| ERK1 | No inhibition | 2819.00 | ND |
| ERK2/MAPK1 /P42MAPK | No inhibition | 4392.00 | ND |
| FAK/PTK2 | No inhibition | 9.54 | ND |
| FER | No inhibition | 1.51 | ND |
| FES/FPS | No inhibition | 2.59 | ND |
| FGFR1 | No inhibition | 1.35 | ND |
| FGFR2 | No inhibition | 4.65 | ND |
| FGFR3 | No inhibition | 15.44 | ND |
| FGR | No inhibition | 0.67 | ND |
| FLT1 | No inhibition | 7.07 | ND |
| FLT3 (CD) | No inhibition | 0.75 | ND |
| FLT4/VEGFR3 | No inhibition | 0.97 | ND |
| FMS | No inhibition | 2.73 | ND |
| FRK/PTK5 | No inhibition | 6.15 | ND |
| FYN | No inhibition | 1.56 | ND |
| GCK/MAP4K2 | No inhibition | 0.93 | ND |
| GRK2 | No inhibition | 892.70 | ND |
| GRK3/ADRBK2 | No inhibition | 594.40 | ND |
| GRK4 | No inhibition | 90.64 | ND |
| GRK5 | No inhibition | 57.04 | ND |
| GRK6 | No inhibition | 20.67 | ND |
| GRK7 | No inhibition | 1.56 | ND |
| GSK3a | No inhibition | 4.87 | ND |
| GSK3β | No inhibition | 8.15 | ND |
| HCK | No inhibition | 1.76 | ND |
| HGK/MAP4K4 | No inhibition | 0.24 | ND |
| HIPK1 | No inhibition | No inhibition | ND |
| HIPK2 | No inhibition | 2003.00 | ND |
| HIPK3 | No inhibition | 1997.00 | ND |
| HIPK4 | No inhibition | 520.90 | ND |
| IGF-1R | No inhibition | 42.66 | ND |
| IKKa/CHUK | No inhibition | 83.00 | ND |
| IKKb | No inhibition | 337.00 | ND |
| IR | No inhibition | 20.70 | ND |
| IRAK1 | No inhibition | 21.03 | ND |
| IRAK4 | No inhibition | 3.87 | ND |
| IRR/INSRR | No inhibition | 9.63 | ND |
| ITK | No inhibition | 5.40 | ND |
| JAK1 | No inhibition | 0.55 | ND |
| JAK3 | No inhibition | 0.15 | ND |
| JNK1a1 | No inhibition | 4439.00 | ND |
| JNK3 | No inhibition | >20000 | ND |
| KDR/VEGFR2 | No inhibition | 5.83 | ND |
| LCK | No inhibition | 6.71 | ND |
| LIMK1 | No inhibition | 2.10 | ND |
| LKB1 | No inhibition | 33.99 | ND |
| LOK/STK10 | No inhibition | 5.08 | ND |
| LRRK2 | No inhibition | 41.22 | ND |
| LYN | No inhibition | 0.41 | ND |
| LYN B | No inhibition | 3.41 | ND |
| MAPKAPK2 | No inhibition | 41.01 | ND |
| MAPKAPK3 | No inhibition | 1531.00 | ND |
| MAPKAPK5/PRAK | No inhibition | 592.30 | ND |
| MARK1 | No inhibition | 0.03 | ND |
| MARK2/PAR-1Ba | No inhibition | 0.03 | ND |
| MARK3 | No inhibition | 0.18 | ND |
| MEK1 | No inhibition | 4.92 | ND |
| MELK | No inhibition | 0.36 | ND |
| MINK/MINK1 | No inhibition | 0.50 | ND |
| MLCK2/MYLK2 | No inhibition | 26.58 | ND |
| MLCK/MYLK | No inhibition | 18.21 | ND |
| MLK1/MAP3K9 | No inhibition | 0.46 | ND |
| MNK2 | No inhibition | 16.95 | ND |
| MRCKα/CDC42BPA | No inhibition | 7.40 | ND |
| MRCKβ/CDC42BPB | No inhibition | 8.70 | ND |
| MSK1/RPS6KA5 | No inhibition | 0.83 | ND |
| MSK2/RPS6KA4 | No inhibition | 3.45 | ND |
| MSSK1/STK23 | No inhibition | 2893.00 | ND |
| MST1/STK4 | No inhibition | 0.55 | ND |
| MST2/STK3 | No inhibition | 2.21 | ND |
| MST3/STK24 | No inhibition | 7.57 | ND |
| MST4 | No inhibition | 5.54 | ND |
| mTOR | No inhibition | ND | 3872.00 |
| MUSK | No inhibition | 16.20 | ND |
| NEK1 | No inhibition | 16.33 | ND |
| NEK3 | No inhibition | >20000 | ND |
| NEK4 | No inhibition | 122.30 | ND |
| NEK6 | No inhibition | No inhibition | ND |
| NLK | No inhibition | 112.40 | ND |
| P38a/MAPK14 | No inhibition | No inhibition | ND |
| P38b/MAPK11 | No inhibition | No inhibition | ND |
| P38d/MAPK13 | No inhibition | 3302.00 | ND |
| P38g/MAPK12 | No inhibition | 5033.00 | ND |
| p70S6K | No inhibition | 1.24 | ND |
| p70S6Kb /RPS6KB2 | No inhibition | 2.44 | ND |
| PAK1 | No inhibition | 3.94 | ND |
| PAK2 | No inhibition | 2.38 | ND |
| PAK3 | No inhibition | 0.40 | ND |
| PAK4 | No inhibition | 54.72 | ND |
| PAK5 | No inhibition | 3.94 | ND |
| PAK6 | No inhibition | 39.18 | ND |
| PASK | No inhibition | 7.85 | ND |
| PBK/TOPK | No inhibition | 111.40 | ND |
| PDGFRa | No inhibition | 0.71 | ND |
| PDGFRb | No inhibition | 1.04 | ND |
| PDK1/PDPK1 | No inhibition | 11.24 | ND |
| PHKg2 | No inhibition | 0.70 | ND |
| PI3K-alpha | >100000 | ND | 528.40 |
| PI3K-beta | >100000 | ND | 1222.00 |
| PI3K-gamma | >100000 | ND | 1497.00 |
| PI3K-delta | >100000 | ND | 659.40 |
| PIM1 | No inhibition | 3.69 | ND |
| PIM2 | No inhibition | 12.77 | ND |
| PKA | No inhibition | 0.97 | ND |
| PKCa | No inhibition | 2.15 | ND |
| PKCbI | No inhibition | 6.14 | ND |
| PKCbII | No inhibition | 0.87 | ND |
| PKCepsilon | No inhibition | 0.03 | ND |
| PKCeta | No inhibition | 1.05 | ND |
| PKCg | No inhibition | 2.15 | ND |
| PKCiota | No inhibition | 15.52 | ND |
| PKCmu/PKD1 | No inhibition | 1.18 | ND |
| PKCnu/PKD3 | No inhibition | 0.68 | ND |
| PKCtheta | No inhibition | 0.80 | ND |
| PKCzeta | No inhibition | 59.85 | ND |
| PKD2/PRKD2 | No inhibition | 1.18 | ND |
| PKG1a | No inhibition | 0.91 | ND |
| PKG1b | No inhibition | 46.82 | ND |
| PKG2/PRKG2 | No inhibition | 3.13 | ND |
| PKN2/PRK2 | No inhibition | 1.98 | ND |
| PLK1 | No inhibition | 466.00 | ND |
| PLK2 | No inhibition | 411.30 | ND |
| PLK3 | No inhibition | 3019.00 | ND |
| PRKX | No inhibition | 0.56 | ND |
| PYK2/PTK2B/FAK2 | No inhibition | 6.41 | ND |
| RET | No inhibition | 1.18 | ND |
| RIPK2 | No inhibition | 685.60 | ND |
| ROCK1 | No inhibition | 0.73 | ND |
| ROCK2/ROKα | No inhibition | 0.57 | ND |
| RON/MST1R | No inhibition | 261.30 | ND |
| ROS/ROS1 | No inhibition | 0.11 | ND |
| RSK1 | No inhibition | 0.09 | ND |
| RSK2 | No inhibition | 0.08 | ND |
| RSK3 | No inhibition | 0.13 | ND |
| RSK4/RPS6KA6 | No inhibition | 0.05 | ND |
| SGK1 (d1-59, S422D) | No inhibition | 6.80 | ND |
| SGK2 | No inhibition | 30.17 | ND |
| SGK3/SGKL | No inhibition | 131.60 | ND |
| SIK2/SNF1LK2/QIK | No inhibition | 45.84 | ND |
| SRPK1 | No inhibition | 62.85 | ND |
| SRPK2 | No inhibition | 307.40 | ND |
| STK22D | No inhibition | 0.02 | ND |
| STK33 | No inhibition | 59.52 | ND |
| SYK | No inhibition | 0.33 | ND |
| TAK1/TAB1/MAP3K7 | No inhibition | 71.38 | ND |
| TAOK1 | No inhibition | 0.42 | ND |
| TAOK2/TAO1 | No inhibition | 3.45 | ND |
| TAOK3/JIK | No inhibition | 1.32 | ND |
| TBK1 | No inhibition | 1.29 | ND |
| TGFRb2 | No inhibition | 10640.00 | ND |
| TIE2/TEK | No inhibition | 105.70 | ND |
| TRKA/NTRK1 | No inhibition | 3.73 | ND |
| TRKB/NTRK2 | No inhibition | 0.06 | ND |
| TRKC | No inhibition | 0.18 | ND |
| TSSK2 | No inhibition | 2.04 | ND |
| TTK | No inhibition | 589.70 | ND |
| TXK | No inhibition | 23.07 | ND |
| TYK1/LTK | No inhibition | 9.75 | ND |
| TYK2 | No inhibition | 0.09 | ND |
| TYRO3/SKY | No inhibition | 5.16 | ND |
| VRK1 | No inhibition | No inhibition | ND |
| WEE1 | No inhibition | 17900.00 | ND |
| WNK2 | No inhibition | 4474.00 | ND |
| WNK3 | No inhibition | 5856.00 | ND |
| YES | No inhibition | 1.65 | ND |
| ZAK/MLTK | No inhibition | 11640.00 | ND |
| ZAP70 | No inhibition | 5.72 | ND |
| ZIPK/DAPK3 | No inhibition | 5.72 | ND |
| PI3K-alpha | No inhibition | ND | 528.40 |
| PI3K-beta | No inhibition | ND | 1222.00 |
| PI3K-gamma | No inhibition | ND | 1497.00 |
| PI3K-delta | No inhibition | ND | 659.40 |
