# Supplementary material for: COTI-2, a novel small molecule that is active against multiple human cancer cell lines in vitro and in vivo
Source: Oncotarget. 2016 May 2;7(27):41363–79. doi: 10.18632/oncotarget.9133 (PMC5173065; doi:10.18632/oncotarget.9133)
Supplement: Supplementary file 3 [file oncotarget-07-41363-s003.docx]

**Supplementary Table 3**. COTI-2 is not a traditional kinase inhibitor as determined by an ATP-competitive KINOMEscan^TM^ kinase assay. COTI-2 was screened against a panel of kinases at a single concentration of 10 µM and the quantitative dissociation constant (K_d_) was determined. The dissociation constant (K_d_) is a measure of binding specificity such that K_d_ > 1 µM is considered relatively weak binding.

| **Kinase** | **K_d_ (nM)** | **COTI-2 Concentration Tested (nM)** |
| --- | --- | --- |
| AAK1 | No Hit | 10,000 |
| ABL1 | No Hit | 10,000 |
| ABL1 (E255K) | No Hit | 10,000 |
| ABL1 (H396P) | No Hit | 10,000 |
| ABL1 (M351T) | No Hit | 10,000 |
| ABL1 (Q252H) | No Hit | 10,000 |
| ABL1 (T315I) | No Hit | 10,000 |
| ABL1 (Y253F) | No Hit | 10,000 |
| ABL2 | No Hit | 10,000 |
| ACVR1 | No Hit | 10,000 |
| ACVR1B | No Hit | 10,000 |
| ACVR2A | No Hit | 10,000 |
| ACVR2B | No Hit | 10,000 |
| ACVRL1 | No Hit | 10,000 |
| ADCK3 | No Hit | 10,000 |
| ADCK4 | No Hit | 10,000 |
| AKT1 | No Hit | 10,000 |
| AKT2 | No Hit | 10,000 |
| AKT3 | No Hit | 10,000 |
| ALK | No Hit | 10,000 |
| AMPK-alpha1 | No Hit | 10,000 |
| AMPL-alpha2 | No Hit | 10,000 |
| AURKA | No Hit | 10,000 |
| AURKB | No Hit | 10,000 |
| AURKC | No Hit | 10,000 |
| AXL | No Hit | 10,000 |
| BIKE | No Hit | 10,000 |
| BLK | No Hit | 10,000 |
| BMPR1A | No Hit | 10,000 |
| BMX | No Hit | 10,000 |
| BRAF | No Hit | 10,000 |
| BFA (V600E) | No Hit | 10,000 |
| BRSK2 | No Hit | 10,000 |
| BTK | No Hit | 10,000 |
| CAMK1 | No Hit | 10,000 |
| CAMK1D | No Hit | 10,000 |
| CAMK1G | No Hit | 10,000 |
| CAMK2A | No Hit | 10,000 |
| CAMK2B | No Hit | 10,000 |
| CAMK2D | No Hit | 10,000 |
| CAMK2G | No Hit | 10,000 |
| CAMKK1 | No Hit | 10,000 |
| CAMKK2 | No Hit | 10,000 |
| CDK2 | No Hit | 10,000 |
| CDK5 | No Hit | 10,000 |
| CLK1 | No Hit | 10,000 |
| CLK2 | No Hit | 10,000 |
| CLK3 | No Hit | 10,000 |
| CLK4 | No Hit | 10,000 |
| CSF1R | No Hit | 10,000 |
| CSK | No Hit | 10,000 |
| CSNK1A1L | No Hit | 10,000 |
| CSNK1D | No Hit | 10,000 |
| CSNK1D | No Hit | 10,000 |
| CSNK1G1 | No Hit | 10,000 |
| CSNK1G2 | No Hit | 10,000 |
| CSNK12A1 | 40,000 | 10,000 |
| CSNK12A2 | 15,900 | 10,000 |
| DAPK2 | No Hit | 10,000 |
| DAPK3 | No Hit | 10,000 |
| DMPK | No Hit | 10,000 |
| EGFR | No Hit | 10,000 |
| EGFR (E746-A750del) | No Hit | 10,000 |
| EGFR (G719C) | No Hit | 10,000 |
| EGFR (G719S) | No Hit | 10,000 |
| EGFR (L747-E749del, A750P) | No Hit | 10,000 |
| EGFR (L747-S752del, P753S) | No Hit | 10,000 |
| EGFR (L747-T751del, Sins) | No Hit | 10,000 |
| EGFR (L858R) | No Hit | 10,000 |
| EGFR (L861Q) | No Hit | 10,000 |
| EGFR (S752-I759del) | No Hit | 10,000 |
| EPHA1 | No Hit | 10,000 |
| EPHA2 | No Hit | 10,000 |
| EPHA3 | No Hit | 10,000 |
| EPHA4 | No Hit | 10,000 |
| EPHA5 | No Hit | 10,000 |
| EPHA6 | No Hit | 10,000 |
| EPHA7 | No Hit | 10,000 |
| EPHA8 | No Hit | 10,000 |
| EPHB | No Hit | 10,000 |
| EPHB1 | No Hit | 10,000 |
| EPHB2 | No Hit | 10,000 |
| EPHB3 | No Hit | 10,000 |
| EPHB4 | No Hit | 10,000 |
| ERBB2 | No Hit | 10,000 |
| ERBB4 | No Hit | 10,000 |
| ERK1 | No Hit | 10,000 |
| ERK2 | No Hit | 10,000 |
| FER | No Hit | 10,000 |
| FES | No Hit | 10,000 |
| FGFR1 | No Hit | 10,000 |
| FGFR2 | No Hit | 10,000 |
| FGFR3 | No Hit | 10,000 |
| FGFR3 (G697C) | No Hit | 10,000 |
| FGFR4 | No Hit | 10,000 |
| FGR | No Hit | 10,000 |
| FLT1 | No Hit | 10,000 |
| FLT3 | No Hit | 10,000 |
| FLT3 (D835H) | No Hit | 10,000 |
| FLT3 (D835Y) | No Hit | 10,000 |
| FLT3 (ITD) | No Hit | 10,000 |
| FLT3 (N841I) | No Hit | 10,000 |
| FLT4 | No Hit | 10,000 |
| FRK | No Hit | 10,000 |
| FYN | No Hit | 10,000 |
| GAK | No Hit | 10,000 |
| GCN2 (Kin.Dom.2.S808G) | No Hit | 10,000 |
| GSK3A | No Hit | 10,000 |
| HCK | No Hit | 10,000 |
| IGF1R | No Hit | 10,000 |
| INSR | No Hit | 10,000 |
| INSRR | No Hit | 10,000 |
| ITK | No Hit | 10,000 |
| JAK1 (Kin.Dom.1) | No Hit | 10,000 |
| JAK2 (Kin.Dom.2) | No Hit | 10,000 |
| JNK1 | 4,540 | 10,000 |
| JNK2 | 3,010 | 10,000 |
| JNK3 | No Hit | 10,000 |
| KIT | No Hit | 10,000 |
| KIT (D816V) | No Hit | 10,000 |
| KIT (V559D) | No Hit | 10,000 |
| KIT (V559D, T670I) | No Hit | 10,000 |
| KIT (V559D, V654A) | No Hit | 10,000 |
| LCK | No Hit | 10,000 |
| LIMK1 | No Hit | 10,000 |
| LIMK2 | No Hit | 10,000 |
| LKB1 | No Hit | 10,000 |
| LOK | No Hit | 10,000 |
| LTK | No Hit | 10,000 |
| LYN | No Hit | 10,000 |
| MAP3K4 | No Hit | 10,000 |
| MAP3K5 | No Hit | 10,000 |
| MAP4K1 | No Hit | 10,000 |
| MAP4K3 | No Hit | 10,000 |
| MAP4K4 | No Hit | 10,000 |
| MAP4K5 | No Hit | 10,000 |
| MARK1 | No Hit | 10,000 |
| MARK2 | No Hit | 10,000 |
| MARK4 | No Hit | 10,000 |
| MERTK | No Hit | 10,000 |
| MET | No Hit | 10,000 |
| MKNK2 | No Hit | 10,000 |
| MLK3 | No Hit | 10,000 |
| MRCKA | No Hit | 10,000 |
| MST4 | No Hit | 10,000 |
| MUSK | No Hit | 10,000 |
| MYLK2 | No Hit | 10,000 |
| NDR2 | No Hit | 10,000 |
| NEK2 | No Hit | 10,000 |
| NEK6 | No Hit | 10,000 |
| NEK7 | No Hit | 10,000 |
| NEK9 | No Hit | 10,000 |
| NLK | No Hit | 10,000 |
| p38-alpha | 4,290 | 10,000 |
| p38-beta | No Hit | 10,000 |
| p38-gamma | No Hit | 10,000 |
| PAK1 | No Hit | 10,000 |
| PAK2 | No Hit | 10,000 |
| PAK3 | No Hit | 10,000 |
| PAK4 | No Hit | 10,000 |
| PAK6 | No Hit | 10,000 |
| PAK7/PAK5 | No Hit | 10,000 |
| PCTK1 | 1,890 | 10,000 |
| PDGFRA | No Hit | 10,000 |
| PDGFRB | No Hit | 10,000 |
| PDPK1 | No Hit | 10,000 |
| PHKG1 | No Hit | 10,000 |
| PHKG2 | No Hit | 10,000 |
| PIM1 | No Hit | 10,000 |
| PIM2 | No Hit | 10,000 |
| PKAC-alpha | No Hit | 10,000 |
| PKAC-beta | No Hit | 10,000 |
| PKMYT1 | No Hit | 10,000 |
| PLK1 | No Hit | 10,000 |
| PLK3 | No Hit | 10,000 |
| PLK4 | 1,250 | 10,000 |
| PRKCH | No Hit | 10,000 |
| PRKG1 | No Hit | 10,000 |
| PRKG2 | No Hit | 10,000 |
| PRKX | No Hit | 10,000 |
| PTK2 | No Hit | 10,000 |
| PTK2B | No Hit | 10,000 |
| PTK6 | No Hit | 10,000 |
| QIK | No Hit | 10,000 |
| RAF1 | No Hit | 10,000 |
| RET | No Hit | 10,000 |
| RIPK2 | No Hit | 10,000 |
| ROS1 | No Hit | 10,000 |
| RPSCKA2 (Kin.Dom.1) | No Hit | 10,000 |
| RPSCKA3 (Kin.Dom.1) | No Hit | 10,000 |
| RPSCKA4 (Kin.Dom.1) | No Hit | 10,000 |
| RPSCKA5 (Kin.Dom.1) | No Hit | 10,000 |
| RPSCKA6 (Kin.Dom.1) | No Hit | 10,000 |
| SLK | No Hit | 10,000 |
| SRC | No Hit | 10,000 |
| SRPK1 | No Hit | 10,000 |
| STK16 | No Hit | 10,000 |
| STK17A | No Hit | 10,000 |
| STK17B | No Hit | 10,000 |
| STK24 | No Hit | 10,000 |
| STK25 | No Hit | 10,000 |
| STK3 | 1,730 | 10,000 |
| STK33 | No Hit | 10,000 |
| STK36 | No Hit | 10,000 |
| STK4 | No Hit | 10,000 |
| SYK | No Hit | 10,000 |
| TESK1 | No Hit | 10,000 |
| TGFBR1 | No Hit | 10,000 |
| TIE2 | No Hit | 10,000 |
| TLK1 | No Hit | 10,000 |
| TLK2 | No Hit | 10,000 |
| TNIK | No Hit | 10,000 |
| TNK1 | No Hit | 10,000 |
| TNK2 | No Hit | 10,000 |
| TNNI3K | No Hit | 10,000 |
| TRKA | No Hit | 10,000 |
| TRKB | No Hit | 10,000 |
| TRKC | No Hit | 10,000 |
| TTK | No Hit | 10,000 |
| TXK | No Hit | 10,000 |
| TYK2 (Kin.Dom.2) | No Hit | 10,000 |
| TYRO3 | No Hit | 10,000 |
| VEGFR2 | No Hit | 10,000 |
| YANK2 | No Hit | 10,000 |
| YANK3 | No Hit | 10,000 |
| YES | No Hit | 10,000 |
| ZAP70 | No Hit | 10,000 |
